# Validation of the Weibull-derived Gini coefficient for quantifying the inequality of the aboveground biomass distribution using 2815 culms from 50 bamboo species

**DOI:** 10.3389/fpls.2026.1820609

**Published:** 2026-06-30

**Authors:** Wen Gu, Peijian Shi, Guohua Liu, Christian Frølund Damgaard, Karl J. Niklas

**Affiliations:** 1 Bamboo Research Institute, College of Forestry and Grassland, Nanjing Forestry University, Nanjing, China; 2 Department of Ecoscience, Aarhus University, Aarhus, Denmark; 3 School of Integrative Plant Science, Cornell University, Ithaca, NY, United States

**Keywords:** aboveground fresh mass, gamma distribution, Lorenz curve, size distribution, Weibull distribution

## Abstract

The Gini coefficient (GC), derived from the Lorenz curve in economics, has been widely applied to quantify the inequality in biological size distributions. Although the GC can be theoretically obtained from distribution functions, previous studies have not cross-validated the distribution-derived GC with empirically determined GC values for important traits such as the aboveground biomass of plants. Here, we used aboveground fresh mass (FM) of >2800 culms from 50 bamboo species with 29−142 culms per species to test whether the FM, as a representative of aboveground biomass, adheres to a normal, log-normal, two-parameter Gamma, or two-parameter Weibull distribution, and compared the distribution-derived GC with the observed GC calculated using the polygon method (denoted as *G_P_*). The Gamma and Weibull distributions provided better fits for most species compared to the normal and log-normal distributions. However, the regression between the Weibull-derived GC and *G_P_* was stronger and more robust than that between the Gamma-derived GC and *G_P_*. This cross-validation between the theoretical and observed Gini coefficients confirms that the Weibull distribution is the best fit for bamboo FM data, and provides a validated inequality measure (i.e., the Weibull-derived GC) that may be extended to other clonal plants or monocultures exhibiting determinate growth (e.g., certain herbaceous crops), but further validation is needed for species with different life histories, particularly those with indeterminate growth.

## 1 Introduction

In forest ecosystems, aboveground biomass varies considerably among individual plants due to the combined effects of biotic and abiotic factors (Yuan et al., 2019; Chen et al., 2021). Biotic drivers include intra− and interspecific competition for resources such as light, water, and nutrients; abiotic influences encompass climate, topography, and soil fertility. This heterogeneity in plant size is not merely a statistical outcome but holds significant ecological and functional importance. Larger individuals, which often dominate resource capture, constitute the primary carbon sinks within stands and substantially contribute to carbon sequestration and forest productivity (Esquivel-Muelbert et al., 2025). Conversely, smaller plants can enhance overall stand production and resource−use efficiency by efficiently utilizing residual ecological niches such as understory light gaps, a process influenced by adaptive growth strategies under shading (Fransson et al., 2021). The study of such size hierarchies, which reflect the inequality in resource acquisition and reproductive output among individuals, is a fundamental aspect of plant ecology (Weiner and Solbrig, 1984).

In managed forests, particularly in monoculture plantations or clonal stands, populations often consist of genetically similar modules (e.g., culms in bamboo groves) that may belong to multiple age cohorts (Sandhu et al., 2013). In such systems, the inequality of the size distribution among individuals or modules arises not from age structure but from micro-environmental heterogeneity, slight genetic variation, asymmetric competition, and yearly fluctuations in climate or nutrient availability. This intrinsic size hierarchy is further modulated by planting density, a key management factor. Density-dependent processes, including competition and self-thinning, fundamentally shape the distribution of biomass among individuals (Yoda et al., 1963; Hara, 1986). The self-thinning rule, describing the trajectory of mean plant size with density in crowded stands, has been a central concept in plant population ecology. However, both its theoretical basis and the exact value of its exponent have been subjects of extensive reevaluation and debate (Weller, 1987, 1989). Consequently, accurately quantifying the degree of the inequality in the aboveground biomass distribution is crucial for the plant science. It provides insights into stand dynamics, competition intensity, and resource use efficiency. From a practical standpoint, understanding and managing the inequality of the size distribution is vital for predicting timber yield, assessing stand health, and ensuring the uniformity of wood properties, which directly influences the economic value and processing quality of forest products. Research on conifers, such as *Pinus banksiana*, has demonstrated how the inequality in the size distribution relates to stand development and growth rates (Metsaranta and Lieffers, 2008).

Quantifying the inequality in the size distribution traditionally relies on the metrics derived from direct measurements of individual plants, such as diameter at breast height, ground diameter, or height, which serve as proxies for biomass (Liu et al., 2016). However, estimating aboveground biomass from these dimensional measurements often involves allometric equations calibrated with destructive harvesting. Due to the high cost and labour intensity of harvesting, sample sizes are typically limited, introducing substantial error into biomass estimates and, consequently, into inequality metrics.

**Figure 1:**
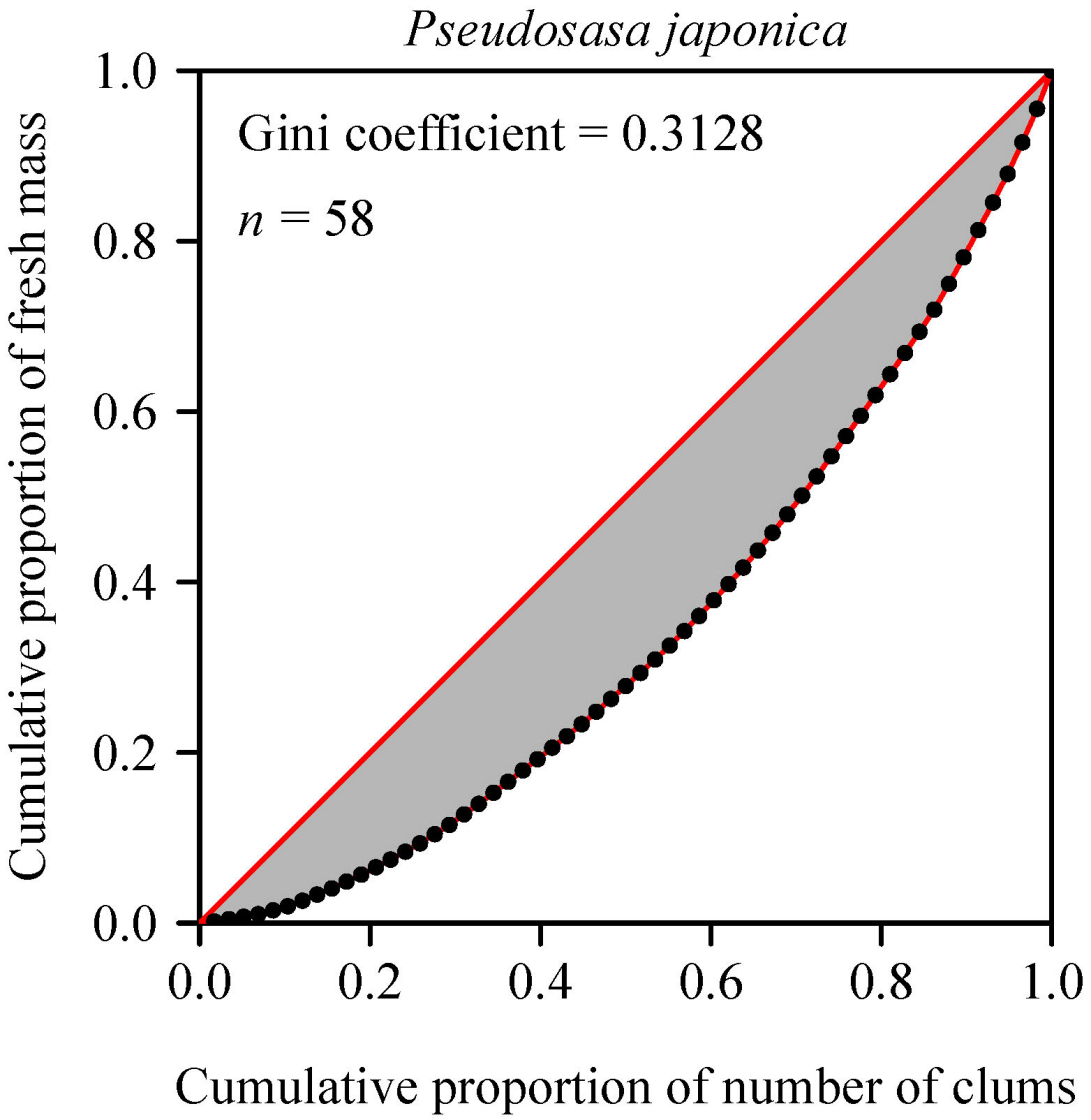
The Lorenz curve of aboveground fresh mass of a representative bamboo species, *Pseudosasa japonica*. The Gini coefficient is equal to twice the area of the shaded region bounded by the line of absolute equality (i.e., the *y = x* straight line) and the Lorenz curve, which was formed by the accumulative proportion of aboveground fresh mass plotted against the accumulative proportion of the number of culms.

A common statistical measure of dispersion, the coefficient of variation (CV), is frequently applied to size data (Shorrocks, 1982). However, the CV is highly sensitive to outliers and does not reflect the shape of the underlying size distribution (e.g., its skewness), a point emphasized in comparisons with other inequality metrics (Bendel et al., 1989). A more robust approach involves characterizing the complete probability distribution of plant size and deriving inequality measures from its parameters. The Gini coefficient, a metric borrowed from economics, is particularly suited for this purpose (Gini, 1912). It is derived from the Lorenz curve, which plots the cumulative proportion of total size (e.g., biomass) against the cumulative proportion of individuals ranked by size (Lorenz, 1905). The Gini coefficient, defined as twice the area between the Lorenz curve and the line of perfect equality, provides a scale-independent measure of the inequality ranging from 0 (perfect equality) to 1 (maximum inequality) (**Figure 1**). Prior studies have established theoretical relationships between the Gini coefficient and the parameters of common statistical distributions, such as the normal, log-normal, Gamma, and Weibull distributions, allowing the inequality to be estimated directly from fitted distribution parameters (Bendel et al., 1989; Lian et al., 2024). This method offers a parsimonious and theoretically grounded alternative, and its application in plant ecology for describing the inequality in the size or fecundity distribution has been formalized (Damgaard and Weiner, 2000). Its validity, however, depends on the empirical agreement between the distribution-derived Gini coefficient and the observed Gini coefficient calculated non-parametrically from the Lorenz curve using methods like the polygon approach, which is reliable when sample sizes are sufficient (Lian et al., 2024; Jiao et al., 2025). Although this cross-validation framework has been successfully applied to leaf area distribution within single bamboo culms, confirming the two-parameter Weibull distribution as a suitable model (Jiao et al., 2025), its applicability to the distribution of aboveground biomass across individual plants or modules, a critical variable in forestry, remains untested. It is unknown whether the Weibull distribution best describes the inequality of the aboveground biomass distribution and whether its derived Gini coefficient can accurately reflect observed inequality in this context. Furthermore, the choice of size metric (e.g., biomass vs. linear dimensions) may affect inequality assessments, as different traits scale allometrically with one another and with overall plant size, thereby altering the shape and interpretation of size distributions (Niklas, 1994).

Recent studies have evaluated probability density functions for modeling diameter distributions in forest stands. For example, Şahin and Dal (2021) compared nine functions for Scots pine stands and found Johnson system of distributions (Johnson’s SB) to be the most successful, with stand characteristics influencing the choice of the best−fitting distribution. Subsequently, Şahin and Ercanlı (2023) evaluated 14 probability density functions in pure and mixed stands and reported that the Rayleigh distribution outperformed traditional functions such as Weibull and Johnson’s SB. However, these studies focused on diameter at breast height, an indirect proxy for biomass, rather than directly on aboveground biomass. Because aboveground fresh mass is more directly related to individual plant size and resource allocation, and because it avoids species−specific allometric scaling errors (Liu et al., 2016), we here extend the distribution−based inequality approach to the actual aboveground biomass of bamboo culms.

Bamboos (subfamily Bambusoideae), prevalent in tropical and subtropical forests, present an ideal model system for investigating the inequality of the size distribution. As major constituents of these ecosystems, bamboos influence understory light environments, nutrient cycling, and provide habitat for a variety of fauna and understory plants. Their growth habit is distinctive: individual culms, emerging from a perennial rhizome system, achieve their full height and diameter within a single growing season, with subsequent growth limited to culm wall thickening. This determinate growth pattern results in stands composed of discrete, easily measurable modules (culms) that achieve their full height and diameter within a single growing season. However, but individual culms within a stand may differ in both age and size (McClure, 1966). Furthermore, bamboo species exhibit an enormous range in mature size, from dwarf species less than 1 meter tall to giant woody species exceeding 30 meters, leading to large interspecific variation in individual biomass (McClure, 1966; Liese and Köhl, 2015). This trait-spectrum makes bamboos excellent for studying size-density relationships, which have been shown to follow the self-thinning rule across species (Liu et al., 2016).

However, research on the inequality of the biomass distribution within bamboo stands, which has direct implications for the uniformity and quality of culms for timber or biomass production, is scarce. To address this gap, we analyzed the aboveground fresh mass (representing the aboveground biomass) of 2815 culms from 50 bamboo species, with 29 to 142 culms per species. We aimed to (1) test whether the observed aboveground fresh mass distributions follow a normal, log-normal, two-parameter Gamma, or two-parameter Weibull distribution, (2) calculate the theoretical Gini coefficients from the parameters of these distributions, and (3) cross-validate these theoretical values against the observed Gini coefficient (calculating using the polygon method) from the empirical Lorenz curve. This study provides a validated methodological framework for quantifying the inequality of the aboveground biomass distribution, offering tools that can enhance the management of bamboo forests and other plantation types (e.g., even−aged monocultures) for improved yield and product uniformity.

## 2 Materials and methods

### 2.1 Bamboo species sampling and data acquisition

Fifty bamboo species growing in bamboo gardens were sampled from (i) Jiangdou, Yangzhou City, Jiangsu Province, (ii) Jurong, Zhenjiang City, Jiangsu Province, and (ii) Anji, Huzhou City, Zhejiang Province of China from May to October in 2014. The area of the sampling quadrat for each species ranged between 0.25 m^2^ and 900 m^2^, where at least 150 culms grew (Liu et al., 2016). Around the center of each quadrat, 29–142 culms were excised at ground level. The total aboveground fresh mass for each culm was weighed within one hour. In total, we measured the aboveground fresh mass of 2815 culms. The species name, quadrat geographical coordinates, quadrat size, and culm number information were tabulated in **Supplementary Table 1**, the data of the aboveground fresh mass of the 50 bamboo species were tabulated in **Supplementary Table 2**.

Although the number of culms sampled per species ranged from 29 to 142 with a median of 50, the sample size for each species was determined primarily by practical constraints related to the loss of fresh mass after cutting, rather than by a purely statistical rule. For small, herbaceous bamboo species, individual fresh mass is low and desiccation is rapid; we therefore sampled a relatively large number of culms for such species to ensure adequate representation. For larger, woody bamboo species, individual fresh mass is much higher and water loss is proportionally smaller within one hour; for these, a sample of 30 to 50 culms is sufficient to obtain stable parameter estimates. Importantly, 47 of the 50 species had 30 or more culms, and the remaining three species had 29 culms each, only marginally below the conventional “large sample” threshold (*n* ≥ 30) commonly used in parametric distribution fitting. We recognize that larger sample sizes would always be desirable, but the substantial effort required to harvest and weigh fresh culms within a one-hour window to prevent water loss made our sample sizes a practical tradeoff. We therefore consider that the observed variation in sample size across species is unlikely to bias the estimation of distribution parameters or the subsequent cross−validation of Gini coefficients, given that nearly all species meet or approach the conventional threshold for stable parametric fitting. We note that quadrat size and culm density varied among species, reflecting the natural inverse relationship between adult bamboo size and stand density (Liu et al., 2016). All quadrats were selected from fully stocked stands with minimal anthropogenic disturbance, and the climatic and soil conditions were similar across the three sampling sites. Therefore, the observed variation in quadrat size and density does not confound the species−level comparisons of size inequality.

### 2.2 Functions for describing the distribution of aboveground fresh mass

We used four density functions (i.e., the normal, log-normal, two-parameter Gamma and two-parameter Weibull distributions, i.e., Equations 1–4) to describe the total aboveground fresh mass per culm (*x*) for each bamboo species:

The normal distribution, denoted as *f_N_*(*x*), takes the form:

(1) $$f_{N}\left(x\right)=\frac{1}{\sqrt{2\pi }\sigma }\exp\left[-\frac{{\left(x-\mu \right)}^{2}}{2\sigma^{2}}\right]$$

where *μ* and *σ* represent the mean and the standard deviation of *x*, respectively.

The log-normal distribution, denoted as *f_L_*(*x*), takes the form:

(2) $$f_{L}\left(x\right)=\frac{1}{\sqrt{2\pi }\sigma_{\log}x}\exp\left\{-\frac{{\left\{\log\left(x\right)-\mu_{\log}\right\}}^{2}}{2\sigma_{\log}^{2}}\right\}$$

where *μ*_log_ and *σ*_log_ represent the mean and the standard deviation of the log(*x*); *x* > 0.

The two-parameter Gamma distribution, denoted as *f_G_*(*x*), takes the form:

(3) $$f_{G}\left(x\right)=\frac{1}{b^{k}\text{ Γ}\left(k\right)}x^{k-1}\exp\left(-\frac{x}{b}\right)$$

where *k* > 0 and *b* > 0 represent the shape parameter and scale parameter, respectively; *x* > 0; Γ(*k*) denotes the gamma function, which takes the form as $\int_{0}^{\infty }t^{k-1}e^{-t}dt$.

The two-parameter Weibull distribution (Weibull, 1951), denoted as *f_W_*(*x*), takes the form:

(4) $$f_{W}\left(x\right)=\frac{\alpha }{\beta }{\left(\frac{x}{\beta }\right)}^{\alpha -1}\exp\left[-{\left(\frac{x}{\beta }\right)}^{\alpha }\right]$$

where *α* > 0 and *β* > 0 represent the shape parameter and scale parameter, respectively; *x* > 0. The numerical value of *α* determines the skewness of distribution: (i) *α <* 3.6, a right-skewed distribution is indicated; (ii) *α* > 3.6, a left-skewed distribution is indicated; and (iii) *α* = 3.6, a symmetrical distribution is indicated (Murthy et al., 2004).

### 2.3 Statistical inference for the distributions

For the normal and log-normal distributions, sample statistics were used to estimate the parameters of their distributions, i.e., the mean $\left(\hat{\mu }=\bar{x_{\text{obs}}}\right)$ and standard deviation $\left(\hat{\sigma }=s_{x_{\text{obs}}}\right)$ of the observations (i.e., *x*_obs_) of total aboveground fresh mass per culm determined the normal distribution, and the mean $\left({\hat{\mu }}_{\log}=\bar{\log\left(x_{\text{obs}}\right)}\right)$ and standard deviation $\left({\hat{\sigma }}_{\log}=s_{\log\left(x_{\text{obs}}\right)}\right)$ of the log-transformation of the observations of total aboveground fresh mass per culm determined the log-normal distribution.

For the two-parameter Gamma and Weibull distributions, parameters were estimated using the maximum likelihood method for each species, which is equivalent to minimize the negative logarithm of the likelihood:

(5) $$-\log\left(\text{Likelihood}\right)=-\sum_{i=1}^{n}\log f\left(x_{\text{obs, }i}\right)$$

which was carried out by the “mle2” function in the “bbmle” package (version 1.0.25.1; Bolker, 2008) based on R (version 4.3.1; R Core Team, 2023). To compare the goodness−of−fit between the two−parameter Gamma and Weibull distributions across the 50 bamboo species, the negative log−likelihood values (Equation 5) obtained for each species were paired by species. The normality of the pairwise differences was assessed using the Shapiro−Wilk test (Royston, 1995). If the normality assumption was met, a paired *t*-test was employed; otherwise, the non-parametric Wilcoxon signed-rank test was used (Wilcoxon, 1945). This two−step procedure ensures robust inference regardless of the distribution of the differences.

The validity of the four distributions was tested using two distinct statistical tests. For the normal and log-normal distributions, the Shapiro-Wilk test (Royston, 1995) was used to test the normality and log-normality of the observed total aboveground fresh mass. For the two-parameter Gamma and Weibull distributions, the Kolmogorov-Smirnov (K-S) test (Schröer and Trenkler, 1995) was used to quantify the goodness-of-fit by comparing the empirical and theoretical cumulative distributions.

### 2.4 Gini coefficients derived from the four distributions

Lorenz (1905) proposed a curve plotting the accumulative percentage of household income against the accumulative percentage of the number of households to reflect the inequality of the household income distribution, which is referred to as the Lorenz curve. The Gini coefficient (GC; Gini, 1912) equals twice the area formed by the Lorenz curve and the line of absolute equality (aligned with the *y = x* straight line). In the present study, we used the Lorenz curve to describe the accumulative proportion of aboveground fresh mass against the accumulative proportion of the number of culms for each species, and calculated the GC as two twice area of the polygon formed by Lorenz curve and the line of absolute equality (**Figure 1**). Because there were 29–142 culms for each species, the GC calculated using the polygon method can be regarded as the ‘observed’ GC, which is denoted as *G_P_* for convenience hereinafter.

Let *F*(*x*) be the cumulative distribution function of the aboveground fresh mass per culm (*x*). It is apparent that an equation describing the Lorenz curve (denoted as the Lorenz function) can be regarded as the quantile function of *F*(*x*). The Lorenz function, *L*(*p*), where *p* represents the cumulative proportion of the number of culms (sorted by the ascending aboveground fresh mass sequence) ranging between 0 and 1, which equals (Gastwirth, 1971; Damgaard and Weiner, 2000; Lian et al., 2024):

(6) $$L\left(p\right)=\mu^{-1}\int_{0}^{p}F^{-1}\left(q\right)dq$$

where *μ* represent the population mean of *x*, equaling $\int_{0}^{\infty }xf\left(x\right)dx$; *q* is the quantile ranging between 0 and 1, and *F*^−1^(*q*) is the quantile function of *F*(*x*). Based on Equation 6, the GC can be written as (Bendel et al., 1989; Lian et al., 2024):

(7) $$\text{GC}=1-2\int_{0}^{1}L\left(p\right)dp=1-2\mu^{-1}\int_{0}^{1}\left(\int_{0}^{p}F^{-1}\left(q\right)dq\right)dp$$

Substituting Equations 1–7, we can obtain the theoretical values of GC corresponding to the normal, lognormal, two-parameter Gamma, and two-parameter Weibull distributions, which were represented by *G_N_*, *G_L_*, *G_G_* and *G_W_* (Equations 8−11), respectively (Bendel et al., 1989; Lian et al., 2024):

The normal-derived GC takes the form:

(8) $$G_{N}=\frac{\sigma }{\mu \sqrt{\pi }}$$

where *μ* and *σ* represent the mean and the standard deviation of the aboveground fresh mass per species, respectively.

The log-normal-derived GC takes the form:

(9) $$G_{L}=2\text{Φ}\left(\frac{\sigma_{\log}}{\sqrt{2}}\right)-1$$

where *σ*_log_ represents the standard deviation of the log-transform of the aboveground fresh mass per species; Φ(·) is the cumulative distribution function of the standard normal distribution.

The Gamma-derived GC takes the form:

(10) $$G_{G}=\frac{\text{Γ}\left(k+0.5\right)}{\sqrt{\pi }\;\text{Γ}\left(k+1\right)}$$

where *k* the shape parameter of the two-parameter Gamma distribution.

The Weibull-derived GC takes the form:

(11) $$G_{W}=1-2^{-\frac{1}{\alpha }}$$

where *α* is the shape parameter of the two-parameter Weibull distribution.

The method of maximum likelihood produced the estimates of the parameters of the two-parameter Gamma and two-parameter Weibull distributions, so the *G_G_* and *G_W_* were calculated based on those estimated parameters. It should be noted that the theoretical Gini coefficients were calculated from the parameters of the fitted distributions, whereas the empirical Gini coefficients were obtained directly from the raw data using the non-parametric polygon method. These two measures are mathematically independent; their comparison serves to evaluate how well the assumed distributions capture the observed inequality in aboveground fresh mass, rather than to derive one from the other.

For the distributions that passed the statistical tests of significance (in the case of most culms), the linear regression (with intercept = 0) between the theoretical and observed Gini coefficients (e.g., *G_G_* versus *G_P_*, and *G_W_* versus *G_P_*) was used to test whether there was an isometric scaling relationship:

(12) y=cx

where *y* and *x* represent the theoretical and observed GCs, and *c* is the proportionality coefficient. In theory, the slope of Equation 12 equals unity. Reduced major axis (RMA) protocols (Niklas, 1994; Quinn and Keough, 2002) were used to estimate the slope, and the bootstrap percentile method (Efron and Tibshirani, 1993; Sandhu et al., 2011) was used to calculate the 95% confidence interval (CI) of the slope. When the 95% confidence intervals of the slopes for both regressions included unity, indicating no significant deviation from the expected isometric relationship, the coefficient of determination (*r*^2^) was used as a criterion to determine which distribution provided a better fit: a higher *r*^2^ value indicated superior performance. In addition, the root-mean-square error (RMSE) in RMA was used to reflect the goodness of fit of, which takes the form:

(13) $$\text{RMSE}=\sqrt{\frac{\text{TEA}}{N}}=\sqrt{\frac{\sum_{j=1}^{N}{\left(y_{j}-{\hat{y}}_{j}\right)}^{2}/\left(2\left|\hat{b}\right|\right)}{N}}$$

where TEA represents the total error area of the triangles formed by the data points (observations) and the regression line (Quinn and Keough, 2002); *y_j_* and ${\hat{y}}_{j}$ represent the observed and predicted *y*-values of the *j*th bamboo species, respectively; $\hat{b}$ represents the estimated slope; and *N* represents the sample size, i.e., the number of bamboo species in the present study. The above procedure, which integrates confidence interval evaluation, coefficient of determination and RMSE (Equation 13) comparison, was employed as a robust approach to compare distribution-based inequality measures.

In addition, to determine whether it is suitable for using *G_P_* (i.e., the GC calculated using the polygon method) as the observed GC, we carried out the correlation analysis on the *G_P_* and coefficient of variation (CV) in aboveground fresh mass. Prior studies have showed that there should be a correlation between the GC and CV in theory (Shorrocks, 1982; Huang et al., 2023).

All statistical analyses were performed at the species level; that is, for each of the 50 bamboo species, the four probability distributions were fitted separately to its own culm aboveground fresh mass data, and species−specific Gini coefficients were derived accordingly. Although bamboos are clonal plants, previous studies on bamboo populations (Sandhu et al., 2013; Liu et al., 2016, 2020; Qin et al., 2018) have demonstrated that the spatial distribution of culms within a fully stocked stand is essentially random and that self−thinning generates substantial size variation among individuals, which supports treating the sampled culms as quasi−independent observations for the purpose of size distribution analysis. Consequently, the potential non−independence among culms is unlikely to bias the estimation of distribution parameters or the calculated Gini coefficients.

## 3 Results

**Figure 2:**
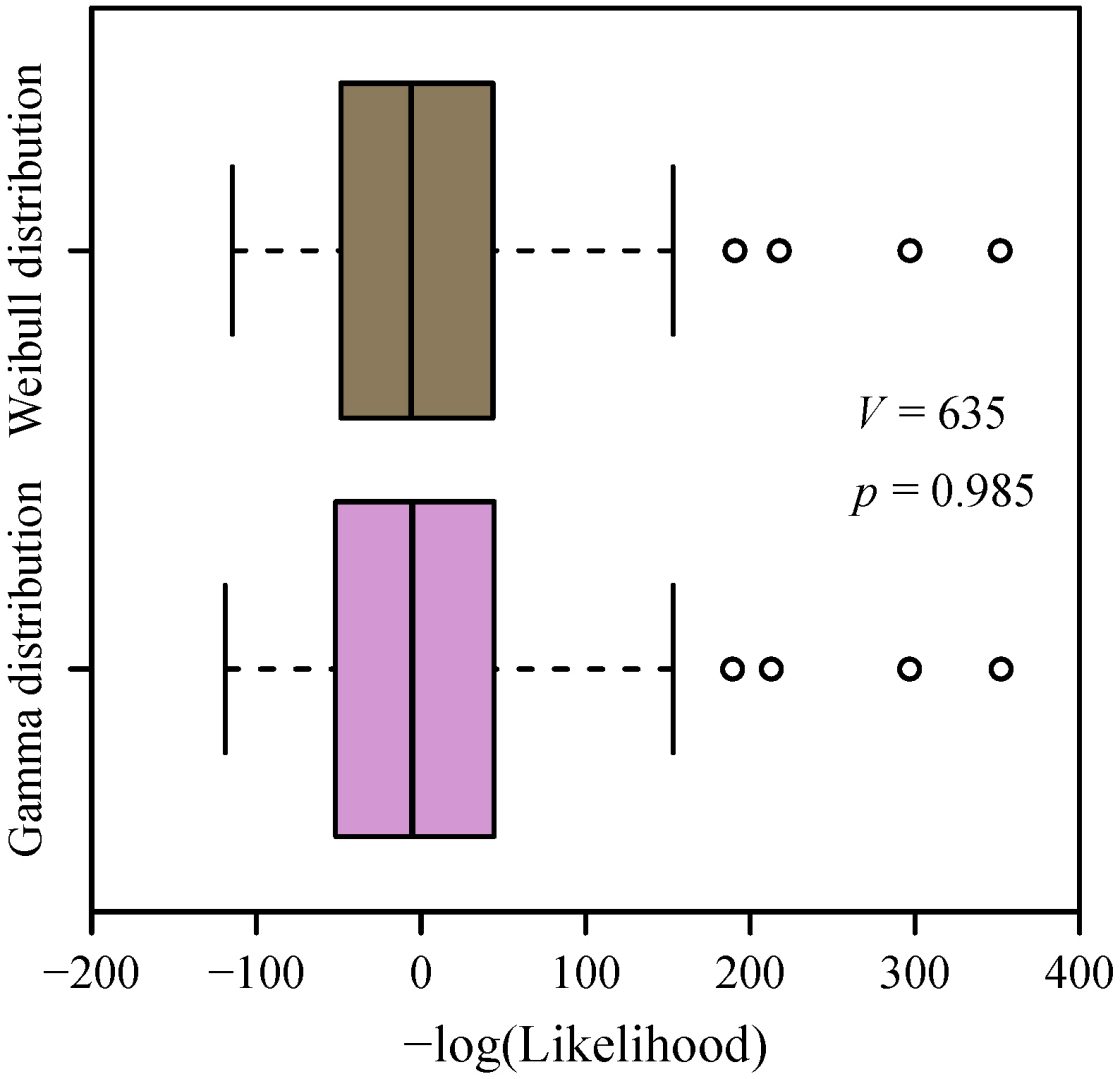
Comparison of the negative natural logarithm of likelihood values of fitting the Gamma and Weibull distributions. The paired non-parametric Wilcoxon signed-rank test was used to test the significance of the difference in the log(likelihood) values between the two distributions.

**Figure 3:**
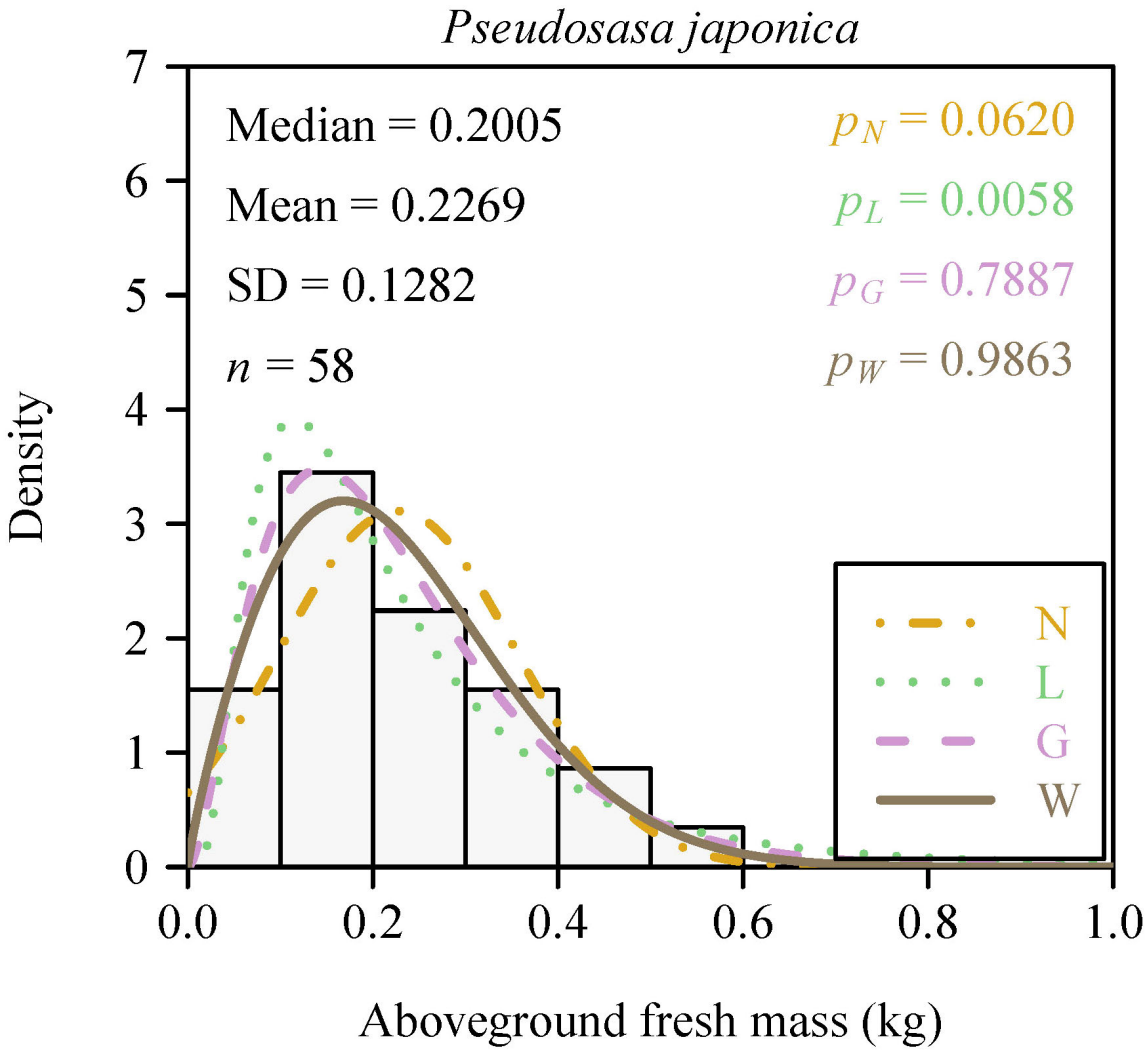
Aboveground fresh mass distribution of a representative bamboo species, *Pseudosasa japonica*. Here, *p_N_* is the probability that the data are consistent with the null hypothesis of a normal distribution; *p_L_* is the probability that the data are consistent with the null hypothesis of a log-normal distribution; *p_G_* is the probability that the data are consistent with the null hypothesis of the two-parameter Gamma distribution; *p_W_* is the probability that the data are consistent with the null hypothesis of the two-parameter Weibull distribution; “SD” is the standard deviation of the data. The colorful curves represent the predicted probability densities by the four distribution functions; “N”, “L”, “G” and “W” represent the normal, log-normal, two-parameter Gamma, and two-parameter Weibull distributions, respectively.

**Figure 4:**
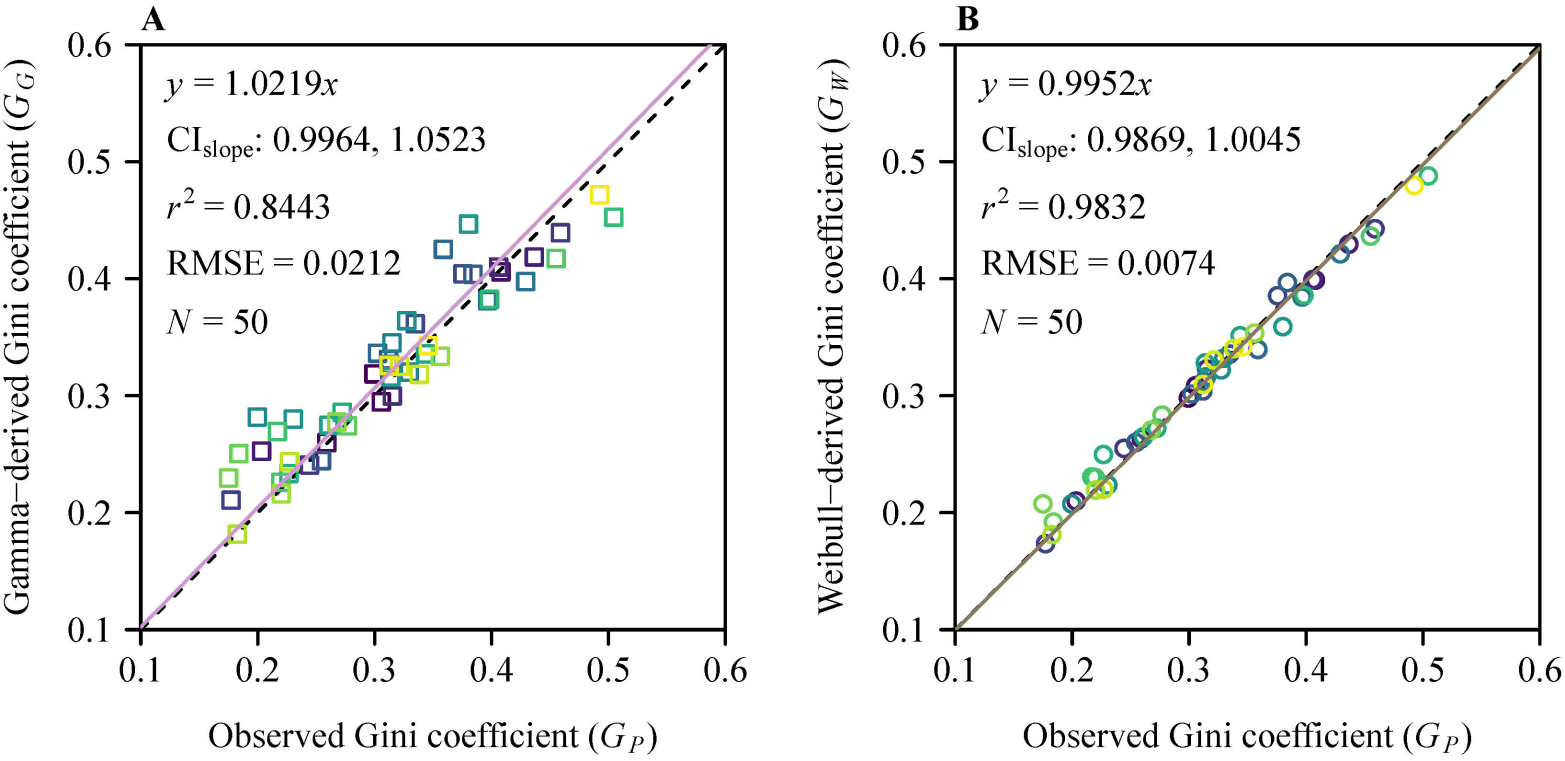
Linear fits to **(A)** the theoretical Gini coefficient of the Gamma distribution (*G_G_*) vs. the observed Gini coefficient calculated using the polygon method (*G_P_*), and **(B)** the theoretical Gini coefficient of the Weibull distribution (*G_W_*) vs. *G_P_*. For each panel, *y* represents *G_G_* in panel **(A)** or *G_W_* in panel **(B)**, and *x* represents *G_P_*; CI_slope_ represents the 95% confidence interval of the slope; *r*^2^ is the coefficient of determination; RMSE is the root-mean-square error of the linear fit; *N* is the sample size, i.e., the number of species.

Thirty percent of culms passed the normality test, and 56% passed the log-normality test. However, 88% of species (44 out of 50) satisfied the two-parameter Gamma distribution, and 92% (46 out of 50) satisfied the two-parameter Weibull distribution (**Supplementary Table 3**). The Shapiro–Wilk test indicated that the paired differences in the negative log−likelihood between the Gamma and Weibull distributions deviated significantly from normality (*W* = 0.851, *p* < 0.001). Therefore, the paired Wilcoxon signed−rank test was used. The test demonstrated no significant difference in the negative log−likelihood between the two distributions (*V* = 635, *p* = 0.985 for a two−sided alternative; **Figure 2**). This result indicated that both distributions performed similarly in fitting the bamboo aboveground fresh mass data. **Figure 3** shows the four predicted distribution curves using the four distribution functions for a representative bamboo species. There was only one estimated Weibull shape parameter greater than 3.6 among the 50 bamboo species; for the remaining 49 bamboo species, the estimated Weibull shape parameter ranged between 1.0359 and 3.467 with a mean (± standard deviation) of 1.9944 (± 0.6428). Therefore, for most bamboo species the predicted Weibull distribution curves are right-skewed. The observed Gini coefficient calculated using the polygon method (*G_P_*​)​ ranged between 0.1750 and 0.5046 with a mean (± standard deviation) of 0.3134 (± 0.0852), which indicated that there was a large variation in the inequality of the aboveground fresh mass across the 50 bamboo species. When comparing theoretical and observed Gini coefficients, the regression of the Gamma-derived Gini coefficient (*G_G_*​) vs. *G_P_*​ and that the Gamma-derived Gini coefficient (*G_W_*​) vs. *G_P_*​​ both yielded a slope whose 95% confidence interval included unity, but the estimated slope of *G_W_*​ vs. *G_P_*​​ was more approximate to unity (**Figure 4**). In addition, the regression of *G_W_*​ vs. *G_P_* was more robust than that of *G_G_*​ vs. *G_P_*, reflected by a greater coefficient (*r*^2^ = 0.9832 vs. *r*^2^ = 0.8443) and a smaller RMSE (RMSE = 0.0074 vs. RMSE = 0.0212). It demonstrated optimal characterization of the intra-specific inequality in the aboveground fresh mass distribution by the two-parameter Weibull distribution.

**Figure 5:**
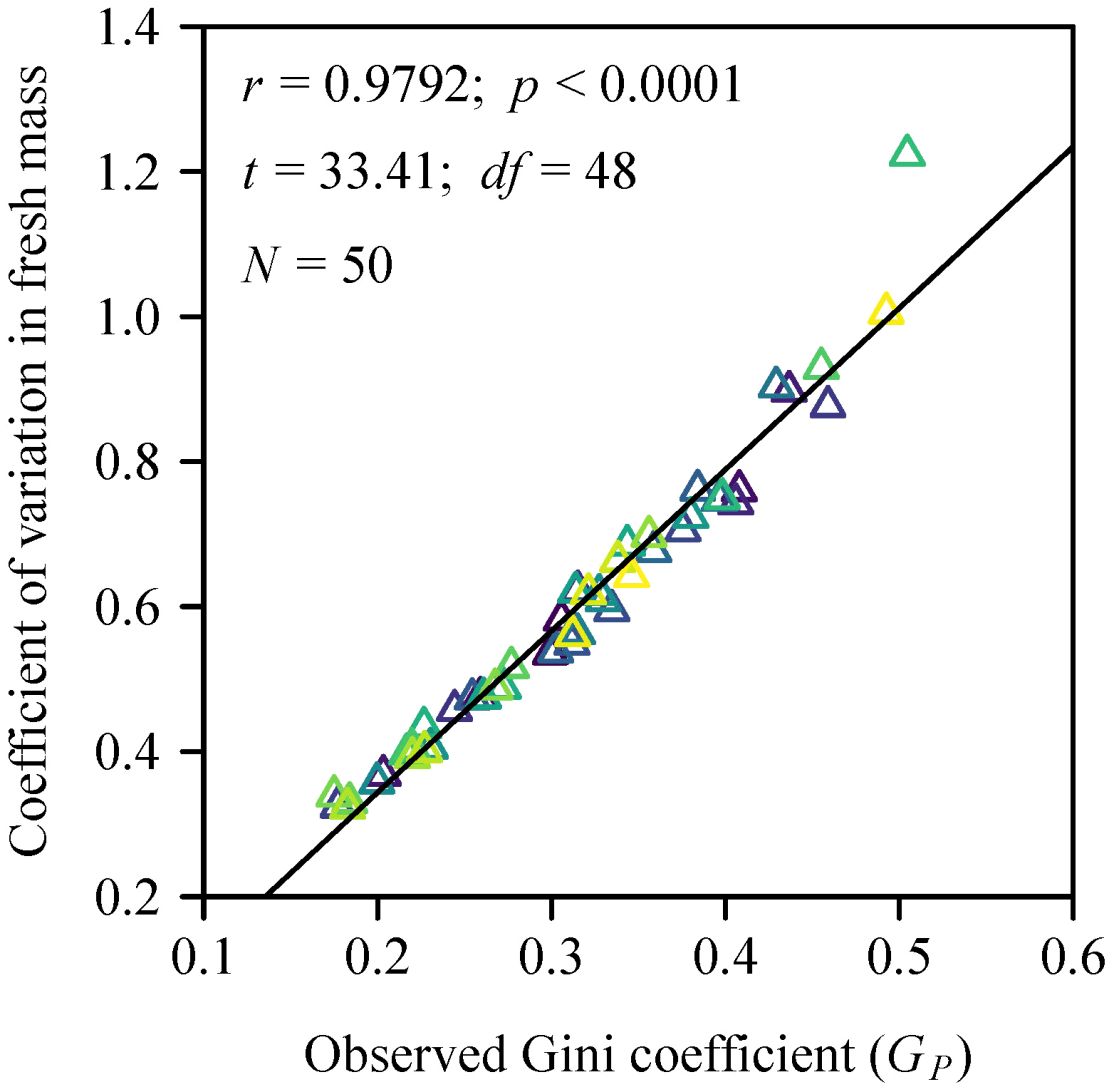
Correlation between the coefficient of variation and the Gini coefficient for aboveground fresh mass. *r* is the correlation coefficient; *p* represents the probability of observing a correlation as strong as the sample result if the true population correlation were zero; the *t*-statistic (with degree of freedom *df* = *N* − 2) is used to test the significance of the correlation coefficient, where *N* represents the sample size, i.e., the number of species.

A significant correlation between *G_P_* and the coefficient of variation in aboveground fresh mass across culms (CV) was confirmed (**Figure 5**), which demonstrated the validity of *G_P_* in serving as observations.

## 4 Discussion

### 4.1 Representativeness of sampled culm aboveground fresh mass

**Figure 6:**
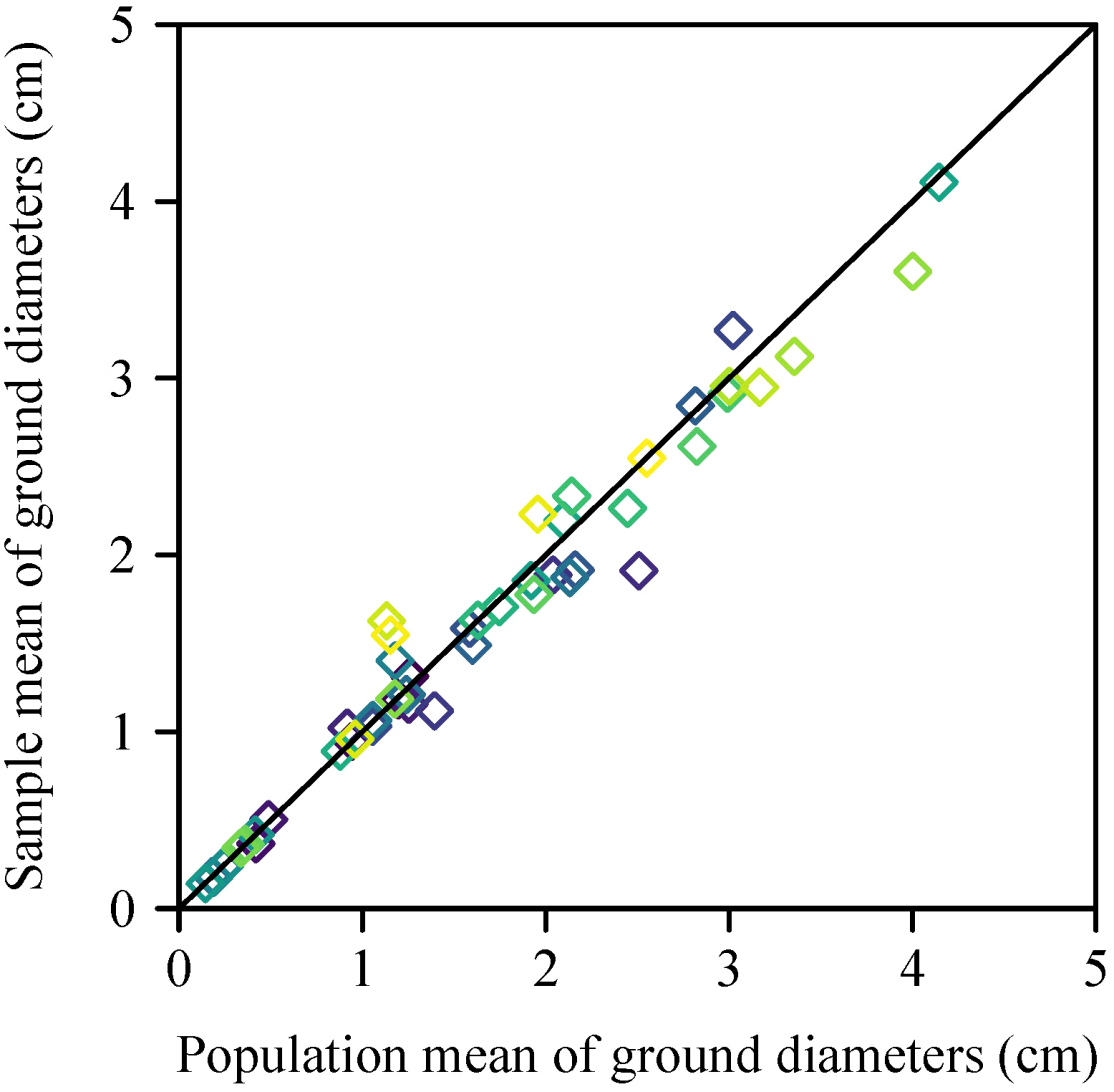
Comparison between the population mean and the sample mean of ground diameter for each species. The population mean of ground diameter denotes the mean of all culms in a quadrat, and the sample mean of ground diameter denotes that the mean of the culms growing around the center of each quadrat sampled for measuring the aboveground biomass.

A critical consideration in this study is whether the aboveground fresh mass (FM) of the sampled culms accurately represents the FM of all culms within the quadrat for each species. To address potential sampling bias, we compared the mean ground diameter (GD) of the sampled culms (those measured for FM around the quadrat center) with the mean GD of all culms within the entire quadrat. GD serves as a reliable proxy for FM due to a strong allometric relationship between the two variables (Liu et al., 2016). A paired *t*-test revealed no significant difference between the population mean GD (all culms) and the sample mean GD (sampled culms) (*t* = 1.2931, *df* = 49, *p* = 0.202; **Figure 6**). This result supports the representativeness of our FM sampling. Furthermore, the lack of significant difference implies an absence of pronounced spatial heterogeneity in culm size within the sampled quadrats. This finding aligns with research on dwarf bamboos, where large individuals were found to be randomly distributed relative to smaller ones at the distance scales investigated, rather than showing spatial aggregation (Qin et al., 2018). The sampled bamboo gardens provided relatively homogeneous edaphic and topographic conditions within each stand. Consequently, sampling culms from around the quadrat center did not systematically bias the measurements towards significantly larger or smaller individuals compared to the overall population. This validation strengthens the reliability of using the polygon method to calculate the observed Gini coefficient (*G_P_*) from our sample as a robust estimate of the true inequality within each stand.

### 4.2 Interspecific variation in observed Gini coefficients

The observed Gini coefficient for aboveground fresh mass (*G_P_*) exhibited considerable variation among the 50 bamboo species, ranging from 0.1750 to 0.5046 (mean ± SD: 0.3134 ± 0.0852). This broad spectrum of inequality can be attributed to the substantial interspecific differences in culm size inherent to the bamboo subfamily. The studied species encompass the full size range of bamboos, classified into woody, shrubby, and dwarf types based on culm height, diameter, and biomass (Liu et al., 2016). These categories reflect fundamental differences in resource allocation, stand architecture, and ecological strategy. Previous analyses of this dataset have showed that mean individual biomass and its variance scale with culm density across species, following the self-thinning rule (assuming that mean culm size negatively scales with spatial density [culm per square meter]) and Taylor’s power law (assuming the mean culm size scales with the variance of culm size), respectively (Liu et al., 2016, 2020). Notably, the variance in individual biomass decreases at a faster rate than the mean biomass as density increases, suggesting that denser stands, often associated with smaller species, tend to have more homogeneous-sized individuals (Liu et al., 2020). Intriguingly, our correlation analysis between *G_P_* and culm spatial density across the 50 species did not yield a significant relationship (*r* = −0.153, *p* = 0.288). This indicates that, at the interspecific level, stand density alone is not the primary driver of the observed variation in the inequality of the aboveground fresh mass distribution. The inequality measure encapsulates the shape of the entire size distribution, influenced by complex interactions of intrinsic growth patterns, genetic architecture, and micro-environmental filtering that vary among species, even at similar densities. The range of *G_P_*​ for the whole-culm aboveground biomass distribution (0.175−0.505) is notably wider than the range reported for the within-culm inequality of the leaf area distribution in *Semiarundinaria densiflora* (0.16−0.28) (Jiao et al., 2025). This discrepancy likely arises from the biological scale of measurement. Leaf area within a single module (culm) is subject to strong apical dominance and developmental constraints, leading to a relatively constrained range of inequality optimized for light capture. In contrast, the aboveground biomass per culm integrates the outcome of long-term competition for space and soil resources among genetically distinct ramets within a clonal system, allowing for greater potential divergence in individual size and thus higher inequality.

Although the Weibull distribution was accepted for 92% of the 50 species, we recognize that species−level variation in size distribution shape (e.g., degree of skewness, kurtosis) might influence the exact *G_W_* value. However, the extremely high coefficient of determination (*r*^2^ = 0.9832) between *G_W_* and *G_P_* indicates that any species−specific deviation is minor and does not affect the overall validity of the Weibull−derived Gini coefficient approach for quantifying inequality of the size distribution. Future studies with larger per−species sample sizes should explore whether species traits such as maximum height or shade tolerance modulate the relationship, but this is beyond the scope of the present study.

### 4.3 Limitations and future research directions

From a biological perspective, the superior performance of the Weibull distribution can be interpreted beyond its statistical fit. In plant canopies, foliage production depends allometrically on branch length and foliage survival follows an age−dependent Weibull distribution (Fleming, 2001). More broadly, the two−parameter Weibull distribution has been shown to be a flexible tool for modeling ecological size or abundance distributions, with its shape parameter reflecting the excess of either rare or abundant species and its scale parameter quantifying the proportion of persistent species in a community (Ulrich et al., 2018). In the context of bamboo aboveground fresh mass, the Weibull distribution naturally captures multiplicative, competition−driven growth processes where larger culms have a disproportionate advantage, which is analogous to a decreasing hazard function in survival analysis (Jiao et al., 2025). This makes the Weibull distribution more ecologically meaningful than the Gamma distribution, which arises from additive exponential events.

This study provides a validated methodology for quantifying the inequality of the aboveground biomass distribution using the Weibull-derived Gini coefficient. However, several limitations should be acknowledged. First, our analysis for each bamboo species is based on data from a single, fully stocked stand at one location. Although this design effectively reflects the interspecific variation, it does not allow us to assess the intraspecific variation in the inequality of the aboveground biomass. Inequality within a species may vary significantly across different environmental conditions, stand ages, or management regimes. Second, although our sampling spanned three sites in two adjacent provinces (Jiangsu and Zhejiang), the climatic and edaphic conditions across these southeastern Chinese sites are relatively similar. This limits the generalizability of our findings to bamboo species growing under remarkedly different abiotic stresses. Future research should focus on widespread and ecologically or economically important bamboo species to test the robustness and predictive power of the Weibull-Gini framework. For instance, *Phyllostachys edulis* (moso bamboo), whose distribution is known to be influenced more by precipitation gradients than by temperature in China (Shi et al., 2020), presents an excellent model system. Investigating moso bamboo stands across its natural latitudinal and precipitation range would reveal whether the inequality in the aboveground biomass distribution varies systematically with key environmental drivers. Such studies could disentangle the effects of genetics, competition intensity, and resource availability on the development of size hierarchies. Furthermore, applying this methodology to even-aged plantations, such as poplar or spruce monocultures, would test its utility beyond bamboo systems for forestry applications aimed at predicting yield uniformity and understanding stand dynamics. However, given the unique growth habit of bamboos, direct extrapolation to species with indeterminate growth or multi−age stands should be made with caution and requires independent verification.

### 4.4 Forest management implications

The validated Weibull-derived Gini coefficient provides a robust metric for quantifying the inequality in the aboveground biomass distribution across bamboo species, offering direct applications for managed bamboo forests and even-aged plantations. In forestry practice, managing stand structure to control the inequality of the size distribution is essential for optimizing timber yield uniformity, stand health, and product quality. The strong relationship between the Weibull-derived Gini coefficient and observed inequality enables managers to assess competition intensity and stand dynamics with minimal destructive sampling. For bamboo plantations, this approach can guide density management decisions to balance biomass production with size homogeneity, which is particularly valuable for timber and bioenergy crops where uniform culm size enhances economic returns. Furthermore, applying this approach to even-aged forest systems, such as poplar or spruce monocultures, could improve yield prediction and silvicultural planning, but this extrapolation requires that the species exhibit determinate or near−determinate growth, which should be verified before application. This methodology supports evidence-based forest management by translating ecological size hierarchies into practical tools for maintaining productivity and uniformity in managed stands.

## Data Availability

The original contributions presented in the study are included in the article/**Supplementary Material**. Further inquiries can be directed to the corresponding authors.
